# The gendered and racialized politics of risk analysis. The case of Frontex

**DOI:** 10.1080/21624887.2019.1644050

**Published:** 2019-07-31

**Authors:** Saskia Stachowitsch, Julia Sachseder

**Affiliations:** a Department of Political Science, University of Vienna; Austrian Institute for International Affairs (oiip), Vienna, Austria; b Department of Political Science, University of Vienna, Vienna, Austria

**Keywords:** Risk analysis, Frontex, EU border security, feminist security studies, postcolonial theory, critical security studies

## Abstract

This article develops a feminist postcolonial approach to risk analysis as an increasingly central security practice in the EU's emerging border management and security regime. For this purpose, we theorize risk analysis as a sense-making practice embedded within colonial power relations. As such, risk analysis problematizes migrants and migration in gendered and racialized ways that make them amenable to border management and other, potentially violent security practices, such as detentions, returns, surveillance, and Search and Rescue. In an exemplary frame analysis of the European Border and Coast Guard Agency's (Frontex) risk analysis report 2016, we show how conceptualizations of risks and solutions by this key actor are informed by gendered and racialized framings of 1) chaos and violence, 2) exploitation of the EU economic and welfare system, and 3) humanitarianism towards 'vulnerable' migrants. With this study, we seek to strengthen feminist and postcolonial interventions into critical security studies on knowledge, power, and expertise. By conceptualizing risk analysis as political, this article pushes critical security theory beyond understandings of security as socially constructed and towards systematically unpacking the meanings of (in)security as implicated in the reproduction of gendered and racialized power relations.

## 1. Introduction

Risk analysis has become a central security practice in today’s increasingly knowledge-based and intelligence-led security regimes. The assessment of risks and vulnerabilities often precedes, accompanies, and follows security operations and thereby shapes how security is defined and enforced and how operations are conducted in terms of resources, practices, infrastructures, actors, technologies, and legitimizations. In the context of EU border security, risk analysis has attained a particularly important role as a depoliticized tool in a controversial policy field characterized by opposing views of member states and the need for efficient resource distribution (Paul 2017). While the problematic implications of the notion of risk and the importance of risk analysis in modern security regimes have been acknowledged and addressed in critical security studies (Aradau and van Munster 2008; De Goede, Simon, and Hoijtink 2014; Petersen 2011), this scholarship has so far not systematically studied the underlying assumptions and power relations that inform risk analysis and how they translate into concrete security practices. This is particularly relevant for addressing the gendered and racialized effects of security practices and their violent consequences for migrants, which have been widely documented and critiqued by feminist and postcolonial scholars in security studies (Basham and Vaughan-Williams 2012; Bosworth, Fili, and Pickering 2017; Carrera and Hernanz 2015; Chisholm and Stachowitsch 2016; Maguire 2010; Pickering 2014).

To grasp the link between risk analysis and gendered and racialized borders, this article conceptualizes risk analysis as a sense-making security practice that problematizes migrants and migration in ways that make them amenable to border management and other security practices, such as detentions, returns, surveillance, and Search and Rescue. From this perspective, risk analysis – like any form of knowledge production – is not a neutral and objective assessment of a reality outside of itself, but co-constitutive of its own subject. It represents a ‘form of power’ (Boswell 2008; Horii 2016, 242; Boswell 2008) that is exercised through defining who/what is considered a threat/risk, who/what is considered in need of protection and which solutions are deemed plausible and necessary. Risk analysis thus effectively defines the meaning of (in)security and its referent objects with real consequences for how security operations are conducted. The framings produced in risk analysis are then continuously inscribed into institutional practices and political decision-making and can thus become locked into contemporary border regimes.

With this conceptualization of risk analysis as political, we specifically take into account the gendered and racialized power relations within which knowledge and expertise on borders, security, threats, and risks are produced. Together with recent work by Howell and Richter-Montpetit (2019) and Wibben (2016), we thereby seek to strengthen feminist and postcolonial interventions into critical security studies because this field has so far sidelined questions of gender and race in the constitution of (in)security. To counter the ‘whitewashing of raciality and coloniality of power and violence’ (p. 2) in security studies, we follow Howell and Richter-Montpetit (2019) in their call for explicitly drawing out how gender and race are constitutive of ‘the human’ that is taken as the basis for various management and security practices (p. 3). We contribute to this debate through a frame analysis (Rein and Schön 1996) of the European Border and Coast Guard Agency’s (Frontex) Risk Analysis Report 2016 (RAR) (Frontex 2016). In this analysis, we examine how gendered and racialized assumptions shape the problematization of migrants and migration by this central EU border security actor. By bringing feminist and postcolonial perspectives to critical security studies, our focus is not only on the way risk analysis is produced and disseminated as a specialized kind of security knowledge but also on the narrative contents, contextualized framings of problems, and legitimizations of solutions. We show how gender and race matter for constructing migrants as risky; for categorizing them as refugees, irregular migrants, terrorists, criminals, or smuggler; for assigning subject positions of protector, protected, and threat; for producing gendered and racialized subjects to be classified and ordered through risk analysis as a tool of colonial governance; and for consequentially sustaining the notion of a superior, progressive, white Europe.

The article proceeds as follows: To provide a conceptual basis for studying risk analysis as a gendered and racialized security practice, we first explain the centrality of Frontex’s risk analysis in EU border security and its power as a meaning-making device. We then develop our theoretical framework by combining practice-oriented and knowledge-focused critical security studies with feminist and postcolonial approaches. After introducing the method of frame analysis (Rein and Schön 1996), we conduct an exemplary case study on the Annual Risk Analysis Report (RAR) 2016 (Frontex 2016). We elucidate how risk analysis problematizes migration and migrants through the gendered and racialized frames of 1) chaos and violence, 2) exploitation of the EU economic and welfare system, and 3) humanitarianism towards ‘vulnerable’ migrants. We conclude by drawing out the merits of a feminist postcolonial approach to risk analysis, offer some preliminary thoughts on how the identified framings are linked to the conduct of security operations, and suggest avenues for future research.

## 2. The case of Frontex’s risk analysis

Frontex forms the centerpiece of an integrated European approach and catalyst for the Europeanization of border controls (Ekelund 2014; Pollak and Slominski 2009). The agency is responsible for the coordination of member states in securing and protecting the external borders of the Schengen Area as well as for guaranteeing free movement within the EU. In 2016, the organization saw its resources and mandate substantially expanded through an institutional reform, which gave Frontex additional power and competences, for instance through the creation of a permanently deployable pool of border guards and equipment or the ability to collect personal data (Frontex 2017). While the agency remains embedded within a network of other actors, and member states in particular have substantial influence on its activities, these transformations indicate that Frontex has evolved into an increasingly powerful institution.

Frontex applies ‘Integrated Border Management’, a comprehensive approach, which comprises various activities, most notably operations on land, sea, and in the air, returns, research and development, and risk analysis (Frontex 2017). Risk analysis is the ‘backbone of integrated border management’ (Paul 2017, 690) and thus a central function of Frontex (Horii 2016; Horsti 2012; Neal 2009). Risk, for the agency, means ‘the magnitude and likelihood of a threat occurring at the external borders’ that ‘will impact on the EU’s internal security, on the security of the external borders’ (Frontex 2012, 12). Risk assessments are conducted within the framework of the common risk analysis model (CIRAM), which was introduced to harmonize member states’ intelligence activities and co-ordinate their operational objectives (Horii 2016, 245f.). Intelligence is at the heart of this process and is defined by Frontex as any information related to risk (threats, vulnerability, impact). Data is mainly obtained from border agencies of the EU member states as well as from Frontex’s own operational activities (Horii 2016), but also through other EU agencies (e.g. EUROPOL) and international organizations, such as the International Organization of Migration. The results are disseminated in various reports. For this article, we focus on the Annual Risk Analysis Report (RAR), which gathers data from 31 EU member state border-control authorities. Bi-monthly analytical reports and open source information are identified as crucial sources (Frontex 2016, 10). The RAR is produced annually and made publicly available via the agency’s website. It is the centerpiece of the risk analysis publications, which consist of quarterly, general and regional, as well as a small number of individual special reports.

Risk analysis directly impacts and defines the rationale and scope of Frontex’s operations in terms of personnel, resources, and practices (Peers, Guild, and Tomkin 2012), which then feed back into risk assessments through data collected, ‘lessons learned’ and ‘best practice’ evaluations (Frontex 2012). A reciprocal cycle between risk analysis and other border security practices – defined by Frontex as the *operational cycle* – is thus established. Risk analysis is the incipient point of this cycle and determines strategic goals, demanded actions, and reactions as well as the agency’s larger program of work. It is followed by the steps of consultations with member states, the compilation of the operational plan, implementation (the conduct of an operation) and, finally, evaluation, which then feeds back into the process of risk analysis and the possible extension of the operation. Risk analysis and other operational practices are therefore co-constitutive, which further inscribes the logics that inform risk analysis into the way security is thought and done, creating potential lock-in effects (Andersson 2012; Horii 2016).

Beyond its importance for Frontex’s own conduct, risk analysis also plays a powerful part in policy-making and resource allocation, for example in the External Border Fund, the Schengen governance package (Horii 2016), the Eurosur Impact Assessment, the Schengen compliance mechanism, and the Internal Security Fund (Paul 2017). Frontex’s risk analysis therefore has far-reaching influence on the conduct of the agency, the member states, and the policy process and further institutionalizes Frontex’s central role in border management (Neal 2009; Paul 2017; Pollak and Slominski 2009). Risk analysis is however not only the technical, managerial baseline for other practices, funding, or policy-making. By making threats and insecurities intelligible, it is also an instrument of the EU’s and the agency’s (in)securitization of borders and migration (Chillaud 2012; Léonard 2011). Through its meaning-making capacities, it problematizes and thereby politicizes migration and border issues. For understanding the so far unstudied role of Frontex’s risk analysis in constructing the meaning of (in)security and the gendered and racialized implications of this process, we suggest a combination of practice-oriented, knowledge-focused approaches in critical security studies and feminist postcolonial perspectives.

## 3. Critical security studies on knowledge and power

Critical security studies provide a useful starting point for the study of risk analysis as a political sense-making practice because they offer a framework for addressing border security institutions, their knowledge-production, and expertise as deeply embedded within societal power structures and ensuing insecurities (Aradau and van Munster 2007; Buzan, Wæver, and de Wilde 1998; c.a.s.e Collective 2006; Hansen 2006; Huysmans 2000). The Paris School or practice approach within this research tradition has particularly addressed the importance of specialized knowledges in border security and migration governance (Amoore 2013; Aradau and van Munster 2007; Côté-Boucher et al. 2014; Johnson et al. 2011; Squire 2015). The understanding of security as practice and as systematically relational (Aradau, Lobo-Guerrero, and van Munster 2008; Bigo 2002, 2014; Huysmans 2006) allows for the conceptualization of risk analysis as a meaning-making security practice that incorporates both discursive and non-discursive elements and is co-constitutive with other security practices, such as enforcement, detention, and profiling. Additionally, these approaches’ emphasis on data gathering, surveillance, expertise, research, and other forms of knowledge production foreground the nexus between power and knowledge (Huysmans 2006; Neal 2009). Thereby, they enable an understanding of risk analysis as a knowledge practice that is linked to power relations reproduced within and by border security regimes (Carmel and Kan 2017; Gundhus 2018; Jeandesboz 2018; Paul 2017).

Critical security studies have thus addressed the problematic implications of the notion of risk and the importance of risk analysis in modern security (Aas and Gundhus 2015; Amoore 2013; Aradau and van Munster 2007; Corry 2012; De Goede, Simon, and Hoijtink 2014; Petersen 2011). However, a systematic study is missing on risk analysis as involved in the reproduction and transformation of asymmetrical intersectional power relations. Critical security studies in genereal have not yet systematically paid attention to how security politics is structured by gender and race inequalities, upon which constructions of (in)security rely and which security practices tend to reproduce (Amir and Kotef 2018; Wibben 2016). Howell and Richter-Montpetit (2019), in their critique of Foucauldian security studies, even argue that the field replicates the ‘whitewashing of raciality and coloniality of modern power and violence’ (p. 2). They contend that this research fails to account for race as fundamental to “notions of ‘the human’ which ‘are always already constituted through the savage and slave other’ (ibid.). Following their call to center racialized and colonial relations, subjects, and spaces, we bring feminist and postcolonial perspectives to practice-oriented and knowledge-focused approaches in critical security studies. Thereby, we capture Frontex’s gendered and racialized sense-making of problems, solutions, and legitimizations and how they contribute to the violent categorization and governing of non-European ‘Others’ as objects of Western security practices.

## 4. Feminist and postcolonial interventions

Feminist and postcolonial approaches in security studies (Barkawi and Laffey 2006; Sjoberg 2010; Chisholm and Stachowitsch 2016; Tickner 1992; Wibben 2011) have not only investigated the gendered and racialized effects of different security policies and practices, but critiqued the concept of security as inherently gendered and racialized. They have done so by drawing on understandings of gender and race as systems of meaning which construct differential value on the basis of perceived dichotomies between masculine/feminine, rational/irrational, civilized/barbarian, us/them, and European/non-European. This has revealed how different gendered and racialized subjects are reproduced through securitization processes (Hansen 2000); how constructions of protector, protected, and threat are underpinning security institutions and governance; and how these reproduce gendered and racialized stereotypes, e.g. of the hypermasculine racialized villain, the white masculine savior, and the feminized victim (Peterson 1992; Young 2003). Postcolonial approaches particularly show how such constructions are bound to Western-centric perspectives on governing (in)security and the continuation of colonial legacies in security regimes (Agathangelou and Ling 2009; Hönke and Müller 2012; Peoples and Vaughan-Williams 2014), especially with regard to border security (Barkawi and Laffey 2006; Vukov and Sheller 2013). These legacies reinstitute colonial notions of Europe and its racialized ‘Other’ into contemporary (EU) border regimes (Andersson 2012; Barbero 2012; Kinnvall 2016; M’charek, Schramm, and Skinner 2014).

With their focus on the sense-making capacities of gender and race, feminist and postcolonial perspectives highlight the relevance of security knowledge and expertise in the reproduction of these colonial inequalities (Wibben 2011). By ‘insist[ing] on the contextual nature of knowledge claims’ (Wibben 2016, 139), they show how such knowledges not only construct security, but also the broader frameworks for making power relations intelligible. This particularly concerns the ways through which subjects become known/knowable and managed/manageable as an integral part of colonial systems of exploitation and violence (Spivak 1993). Knowledge-based practices, such as categorizing, measuring, ‘mapping, visioning, and surveying’ (Vukov and Sheller 2013, 229), thus intersect with violent practices of oppression and discrimination. As such, gendered and racialized constructions of (in)security as sense-making tools can be linked to insecurities and entail violent consequences for migrants, particularly for women and other vulnerable groups (De Jong 2016; Gerard and Pickering 2014) as well as lead to and legitimize racist and sexist security practices (Pickering 2014; Williams 2003). From this perspective, risk analysis with its historical roots in colonial knowledge practices (M’charek, Schramm, and Skinner 2014) can be understood as a (neo)colonial tool of governance that attempts at ordering and governing the non-European ‘Other’ – often in violent ways.

Taken together, feminist and postcolonial approaches contribute a definition of criticality to security studies that puts unequal and (post)colonial power relations at the center of the analysis and importantly link these power relations to security knowledges. This allows us to examine gendering and racialization in risk analysis as processes of categorization and broader sense-making in border security which build upon and reaffirm hierarchical social power relations and are always embedded within the context of (post)coloniality. By bringing these insights to practice-oriented critical security studies, we aim to strengthen synergies between these research agendas which share epistemological, theoretical, and methodological commitments, such as attention to practice, the everyday, non-elite politics, narratives, and knowledges (Hönke and Müller 2012; Wibben 2016). A combined approach pushes critical security theory beyond understandings of security as socially constructed and towards systematically unpacking the meanings of (in)security as implicated in the reproduction of social power relations which are always gendered and racialized.

## 5. Methods and material

Our exemplary case study examines Frontex’s annual risk analysis report for 2016 (Frontex 2016). This report covers the period of the so-called ‘refugee crisis’ that captured the attention of media and political discourses. This highly politicized moment was also loaded with gendered and racialized security narratives and discourses (Gray and Franck 2019) that are core to our research interest. Further, the period of investigation coincides with a large-scale institutional reform of the agency, which significantly expanded its resources and mandate. In a comparative analysis of Frontex’s risk analysis reports from 2012 to 2016 (Stachowitsch, Sachseder, and Binder 2019), we observed that gendered and racialized representations of ‘Otherness’ – together with the overall intensification of constructions of migration as inherently problematic and threatening – increased in quality and quantity in the 2016 report. It therefore is a suitable starting point for drawing out the merits of a feminist and postcolonial approach to risk analysis and for scrutinizing the sense-making work that gender and race do in these reports.

Building on Andrijasevic and Walters' (2010) understanding of border management as ‘problem management’, i.e. reliant on the construction of migration as problematic and the definition of specific problems arising out of migration movements, we conduct a frame analysis (Rein and Schön 1996) of the RAR 2016. Frame analysis enables the highlighting of the salient and selective processes of ‘naming and framing’ with which actors make sense of complex realities by defining problems and then constructing solutions as ‘logical consequences’ (p. 26). Through these processes, ‘stories make the “normative leap” from data to recommendation, from fact to values, from “is” to “ought”’ (ibid.). With this method, we scrutinize the underlying assumptions and perceptions of the RAR 2016 and highlight how risks, threats, problems, and solutions are conceptualized. This allows us to move beyond studying knowledge production in the form of neutral data gathering and interpretation towards grasping the narrative frameworks and contextualized meanings that underpin risk analysis, elucidate the power asymmetries that inform these frames, and study how power relations are reproduced through the often violent ‘solutions’ deduced from these frames.

To identify the dominant frames in the RAR, we adapted Rein and Schön (1996) model to focus on the problem definitions in the report, i.e. the constructions of risks, and what they revealed and obscured. In the first step, we asked what the defined risks were (e.g. irregular crossings, crime, terrorism, etc.) and how they were problematized (e.g. as an issue of economic overstretch, a security problem, a humanitarian crisis, etc.). In an inductive coding process, we identified three dominant frames: 1) *chaos, crisis and violence;* 2) *economic exploitation and welfare overstretch;* and 3) *humanitarianism*. In a second step, we asked which solutions were suggested, i.e. which knowledge, management, and enforcement practices were deduced from the constructed problems. In a third step, we examined how problem-constructions and solutions were gendered and racialized. For this purpose, we looked for depiction of represented subjects, particularly men/women and Europeans/non-Europeans, how they mapped onto constructions of protector, protected, victim, and threat, constructions of ‘Europe’ (European values, identities) and its ‘Other’, and the use of gendered and racialized language, narratives, and images. For the solutions, we specifically asked how they were linked to colonial knowledge and enforcement practices, what the referent objects of these solutions were, and whose security was (not) considered.

While our analysis primarily examines the written text, we acknowledge the importance of images in security contexts (Hansen 2011; Vuori 2010; Williams 2003). We include an exemplary analysis of key images in the RAR, which heavily draws on photographs, maps, graphs, and tables to visualize (some of) its operational activities, migration movements, and immigration numbers. With a particular focus on how gender and race are reproduced through visual representations of men/women, masculinities/femininities, European/non-European in the RAR 2016, we relate the findings to the frames identified in the text analysis to reveal their mutual reinforcement and/or contradictions.

## 6. Analysis of the RAR 2016

The frame analysis shows that the RAR privileges two predominant constructions of risk to the security of the EUropean continent through migration: chaos and violence associated with the quantity and intensity of migration movements; and exploitation of the European welfare system linked to ‘economic migration’. In both cases, gender, race, and colonial legacies have a central role in constructing risk through ‘Otherness’ and function to make these risks intelligible. Through processes of masculinization and racialization, migrants are produced as securitized villains (e.g. terrorists, criminals), unknown and unmanageable crowds, and exploitative subjects (e.g. the economic migrant). In addition to these threat scenarios, the analysis identified a humanitarian frame which produces migrants as both ‘a risk and at risk’ (Andersson 2012; Pallister-Wilkins 2015). This frame makes sense of migrants’ vulnerability through the narrative of masculinist protectionism (Young 2003) which entails the feminization and racialization of migrants. Both risk constructions and humanitarian tropes produce an inextricable connection between the recognition of the ‘Other’ and the construction of the self (Ahmed 2000, 26), which underpin und reproduce colonial relations of power.

### 6.1. Frame 1: chaos, crisis and violence

The first identified frame produces migration as a security risk and creates an exceptional, often militarized threat scenario with frequent referrals to ‘unprecedentedness’ and a sudden need to act, using emotional, alarmist language:

Unlike almost any other year since World War II, the scenes of chaos and the tragic images of those who have lost their lives have sharpened the focus on migration issues*'* (p. 5)

**Figure 1.**
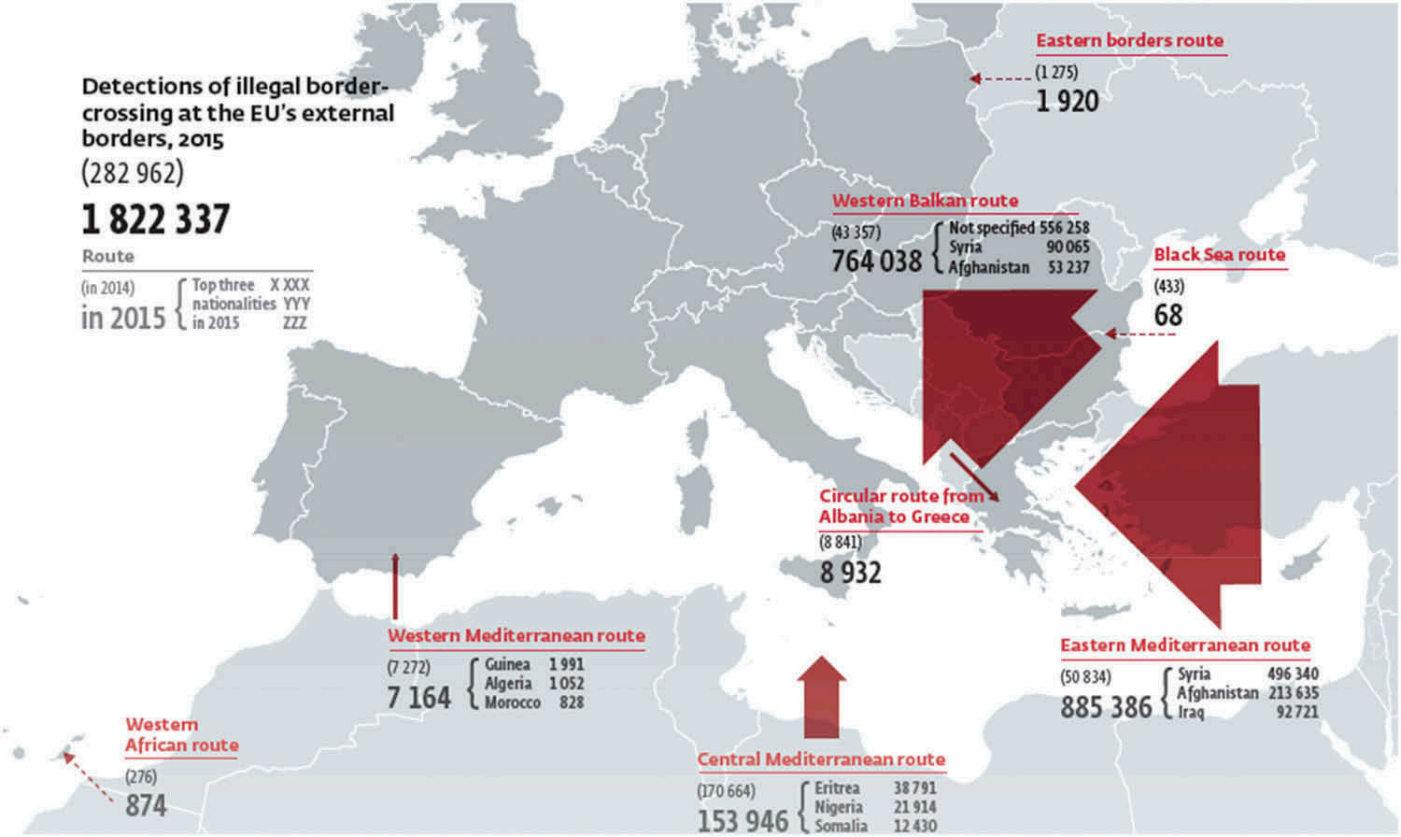
Detections of illegal border-crossings 2015, Frontex 2016, p. 16.

While securitized villains, such as terrorists and criminals feature in this frame, risk is primarily understood as stemming from ‘migratory pressure’, ‘crowds’, and people crossing the border ‘en masse’ (p. 44; ‘intense pressure’, p. 7 and 44; ‘sudden large flows’, p. 44) which is frequently related to the loss of control (p. 6). This framing of being outnumbered and overrun is also prevalent in the imagery, with no less than 18 maps visualizing the EU external borders, sometimes as being overridden by large arrows, or EU member states disappearing behind ever widening circles representing immigration numbers (see Figure 1).

Gender and race have a central role in constructing these risks through, on the one hand, ‘invok[ing] the fantasy of an invasion from the South’ (Andrijasevic 2010, 156) and, on the other, through linking these masses to problematic ‘Other’/racialized masculinities. These constructions can be observed in characterizations of hypermasculine ‘battle-hardened jihadists’ who spread their ‘atrocious modi operandi’ to Europe (p. 39) but, most prominently, in the construction of the ‘people smuggler’ or ‘facilitator’.

The most life-threatening incidents are related to violence of the smugglers against the migrants. Motivated by profits, smugglers increasingly put migrant’s lives at risk. (p. 44)

The latter in particular is characterized through masculinized and racialized attributes and constructed as ‘ruthless’ (Frontex 2016, 39, 44), ‘aggressive’ (p. 44), and ‘profit-making’ (p. 8, 18). He is blamed for ‘tragic deaths’ (p. 5, 26) and ‘violent incidents’ (p. 8) and accused of exploiting the good will of the European Search and Rescue teams. At the same time, the pressure/crisis frame extends the notion of risk to all migrant masculinities by problematizing them as potentially aggressive, violent, unpredictable, and a managerial challenge to Western organizational and law enforcement institutions:

Large numbers of people crossing the border en masse have led to violence requiring public order policing. (p. 8)

Incidents at the border […] have shown that many migrants do not stop when requested to do so by border guards, they do not obey orders of border authorities and are not afraid to engage in physical contact while crossing the border. (p. 45)

Migrants’ ‘unknownness’ and indistinguishability to the Western eye is particularly emphasized as a source of danger and risk. This relates not only to lacking information on who might be a terrorist or criminal but also to insufficient knowledge on countries of origin, migratory routes, etc.:

How to distinguish legitimate asylum-seekers […] from individuals posing a security threat and economic migrants attempting to abuse the system by claiming a false nationality. (p. 5)

Risks are imagined to emanate from gendered and racialized migrants, i.e. ‘single men’ and their different ‘backgrounds and nationalities’ (p. 45). These crowds are unmanageable because they do not conform to Western rationalities and liberal values, including ‘acceptable’ gender norms, which would require male migrants not to endanger ‘their’ women:

[Crowds at the border] gathered people from very different backgrounds and nationalities, rendering the communication of orders and the circulation of basic information difficult. The crowds also mixed young single men with more vulnerable families, including women and children, sometimes purposely put in front of the groups to facilitate their progression. This makes them different from other types of crowds typically managed by law enforcement authorities […]. (p. 45)

Risk is further constituted through migrants’ masculinized agency, i.e. deliberate actions and decisions which render them undeserving because only those who conform to stereotypes of passive victimhood are accepted as legitimate (Pickering 2014). By gendering and racializing the involved actors, resistance against border authorities is categorized as violence and hence presents a security risk (p. 9). These framings draw on sexist and colonialist dichotomies of active/passive and victim/perpetrator which support an ‘us-them’ dichotomy rooted in colonial power relations (Barbero 2012). From this perspective, those who are racialized are ‘always already positioned as enslaved, denied even the right to self-defense, […] subject to gratuitous violence independent of any perceived transgression’ (Howell and Richter-Montpetit 2019, 9). These colonial ascriptions not only relate to migrant subjects but also more broadly to the gendering and racialization of African and Middle Eastern regions as ‘problem-spaces’ (Chamlian 2016). These spaces are characterized as ‘weak’ and ‘failed’ (p. 39) in contrast to Europe as strong and effective, and as leading to the rise of problematic (‘ruthless’, ‘battle-hardened’) masculinities. Such colonial processes of racialization thus place these countries as remote, far away, ‘at the edge of the world’ and in so doing reaffirm the boundaries between Europe and its ‘Other’ (Mbembe 2001).

Another risk in the chaos/crisis frame that foregrounds unknown, unmanaged crowds and is gendered and racialized concerns the discussion of migrants’ poor health status as a risk to Europe and border workforces

In the countries of destination, migration often stretches the capacity of healthcare systems to adapt to the additional demand for health services, and the unfamiliar and changing health profiles and needs. (p. 48)

**Figure 2.**
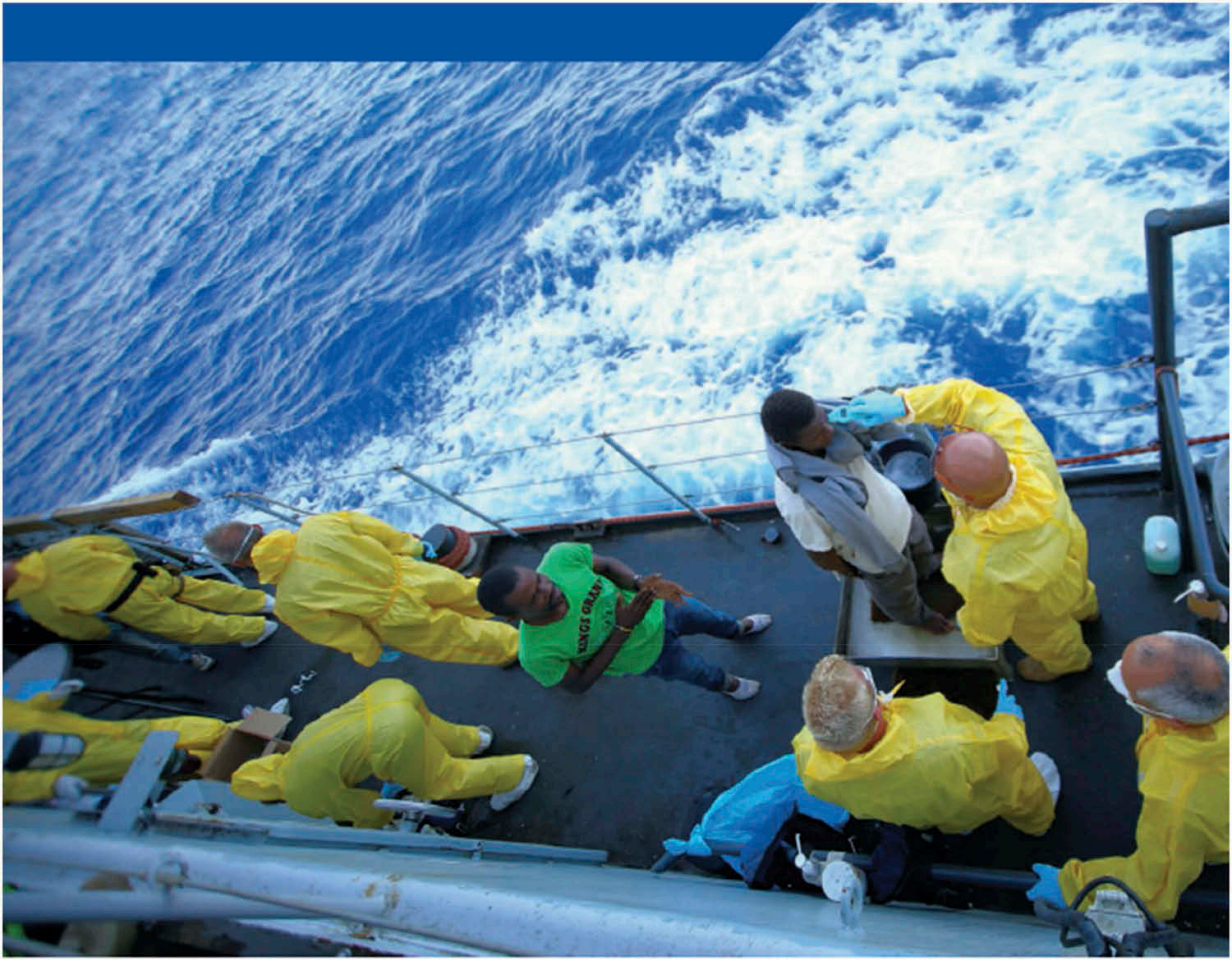
Medical examination, Frontex 2016, p. 48.

The visual analysis underscores this threat scenario: All pictures of rescue missions in which agents interact with migrants show border guards in clinical uniforms. In the picture that accompanies the health chapter (p. 48, see Figure 2), we see a medical examination of black migrants by white officers who are fully clad in protection overalls and breathing masks. Border officers are shown in the colonial role as checking, screening, and examining black bodies and the managing of disease. These narratives and images refer to the realm of global health and the protection against infectious disease which is structured by deep power hierarchies between the Global South and Global North (Pallister-Wilkins 2016). In this context, depictions of Westerners ‘clad in plastic clothing with their faces obscured by masks and goggles have become an easy visual cue for the virus itself’ (ibid.: 507). These notions construct Europe and the imagined homogenous ‘Europeans’ as the principal subjects of protection, while those who suffer from disease or as an effect of postcolonial power relations are ignored (ibid.: 515). In the tradition of colonial humanitarianism (see below), the report does not consider the psychological, mental, and emotional health of potentially traumatized refugees, whilst reproducing colonial understandings of Europe’s ‘Other’ as unclean, diseased, and un-modern and linked to the threat of exploitation and overstretch.

The emphasis on ‘unknownness’ of migrants, including their suspect health status, ‘false declarations’ of nationality, and document fraud, in the above frames is consequential for the proposed solutions which privilege knowledge-based and managerial approaches and lead to the reductionist conclusion that problems are solvable through ‘increased screening, registration’, categorization, information sharing, data gathering and exchange, as well as surveillance (p. 5).

The biometric data of many migrants are missing, which prevents law-enforcement authorities in the EU from effectively using the Eudora for the purpose of preventing, detecting or investigating serious criminal offices or even terrorist offenses. (p. 43)

These knowledge-based approaches and solutions not only build on gendered and racialized constructions of risks but themselves reproduce colonial practices of subjectification, such as fingerprinting, through which the colonizers sought to ‘know’ the colonized (Maguire 2010, 597). As such, they do not only require and produce gendered and racialized bodies but take the colonised body itself as a source of data extraction and knowledge production, e.g. to reconstruct migration routes, identify origin countries, or ‘manage’ disease. Both the management of these bodies and their use as data sources for categorization and ordering purposes are mutually enhancing and reproduce constructions of race, blackness and whiteness, and 'European-ness'. This, in turn, reaffirms the need for colonial ordering of chaotic, unknown ‘Otherness’.

### 6.2. Frame 2: economic exploitation and welfare overstretch

The second central risk construction concerns ‘economic migration’, a framing, which foregrounds economic incentives as motivations for migration and problematizes the exploitation of the EUropean welfare system:

The motivation for migration may vary among individuals but most are believed to be pushed by economic motivations. (p. 41)

Compared to ‘economic’ explanations, issues like war, human rights violations, and human suffering in general are mentioned only sporadically. These constructions of economic migration divert attention from the ‘complex global inequalities’ that underlie the ‘constrained choice’ to migrate (Kmak 2012, 12). Like Kmak (2012) has shown for European immigration law, the RAR produces the ‘economic migrant’ as a rational, but immoral ‘homo oeconomicus’ and thereby supports the presupposition that migrants are different and less deserving than the EU population. What makes these allegedly rational actors immoral greatly hinges on their masculinized and racialized representation as mainly ‘young men’ (p. 41), who ‘tak[e] advantage of the situation’ (p. 8) and are ‘pushed by economic motivations’ (p. 41). They are reified in statements, such as ‘Syrian nationals who *decided* to take the fast air route’ (p. 25), ‘persons *decide* to stay illegally’ (p. 34) or ‘most West African who cannot obtain an EU visa and still *wish* to reach the EU illegally now *opt to* first travel by land’ (p. 41) (all emphasis by authors).

Delays in return also often encourage additional arrivals, because for those unsatisfied with the local economic conditions even a temporary provision of food and shelter combined with a small allowance is an incentives to travel to the EU. (p. 51)

The centrality of this trope is also highlighted by the *only* migrant voice featured in the report:

Some of my friends went to Europe and when they came back, they had money and bought cars for their families. One day I thought […] I should do the same. (p. 20)

In these quotes, migration is caused by ‘dissatisfaction’, the aim to exploit the EU welfare system, and the objective to obtain (even short-term) material gains. These framings build upon and require masculinization and racialization to delegitimize migrants as materialistic, ‘risky’, a potential welfare leech, and undeserving, thereby denying them equal opportunities. Therein, the economic migrant embodies a range of racist stereotypes and anxieties in terms of undermining, exploiting and disrupting the European economic and welfare system. These categorizations not only obscure migrants’ deprived living situations at home but also play out against the background of the welfare state crisis in the EU, in which the question of who has the right to social provisions becomes more and more racialized (Kmak 2012). The problem to Europe’s economic and welfare system is thereby especially identifiable in the body of the racialized economic migrant and thus legitimizes the RAR’s demand for more robust control and enforcement measures, such as border checks, prevention of departures, and returns as a way to ‘decrease incentives for irregular migrants’ (ibid.):

One of the incentives for irregular migrants is the knowledge that the EU’s return system – meant to return irregular migrants or those whose asylum applications are refused – works imperfectly. (p.5)

The intense focus on the ‘imperfect return system’, which is identified as a core problem and also a cause of migration, requires the figure of the economic migrant to justify deportations and detentions. For only if economic incentives are assumed to be the main roots of migration, rather than war, human rights violations, extreme poverty, oppression, and despair, is it credible that returns would be such central answers to the challenges of bordering Europe. Hence, while the prevention of economic migration is framed as a rational bureaucratic response to the problem of economic overstretch and thereby obscures colonial power relations, it requires masculinization and racialization to portray the economic migrant as a threat and thus the protection of the EUropean values and its economy as the paramount goal of border management. In so doing, the RAR legitimizes the forced return mechanisms on colonialist grounds.

### 6.3. Frame 3: humanitarianism as masculinist protectionism

The RAR’s risk construction is two-sided because migrants are not only understood as ‘a risk’ to the EU, but also ‘at risk’ (Andersson 2012; Pallister-Wilkins 2015), with both perspectives intrinsically linked to each other through gendering and racialization. As shown by a growing number of scholars (Aas and Gundhus 2015; Andrijasevic 2010; Pallister-Wilkins 2015, 2016; Perkowski 2018) humanitarianism is an important discourse and practice underpinning and legitimizing modern border regimes. This frame is also dominant in the RAR, which puts ‘saving lives’ (p. 5) and ‘vulnerable’ people (p. 18) as a central rationale for its enforcement measures, such as returns and prevention of departures:

The measures that prevented departures from West Africa to Spain, and that contributed to saving thousands of lives […]. (p.50)

The link between humanitarian and repressive security practices is achieved through the ‘interoperable’ use of key humanitarian concepts, such as ‘vulnerability’ which is employed to refer to penetrable borders (p. 21); vulnerable systems, e.g. for the checking of documents (p. 14); and, finally, to ‘vulnerable’ populations (p. 5). The concept is taken out of its emancipatory context, which stresses unequal ‘distribution of resources and voice/agency’ (Jansson and Eduards 2016, 593) and used to make both managerial concerns and militarized protection appear humanitarian.

As feminist scholars have shown for humanitarian interventions, peacekeeping (Duncanson 2009), and US border protection (Williams 2016), this humanitarianism hinges on masculinist-protectionist representations of gendered and racialized victims, villains, and protectors (Young 2003). In contrast to the masculinization of economic migrants and smugglers as possessing agency, the RAR feminizes some migrants as ‘vulnerable’, naïve and exploitable subjects, making an uninformed decision, unaware of dangers. With a focus on ‘womenandchildren’ (Enloe 1990), these interpretations dispose migrants of agency and rationality and play into the gendered dichotomies of victim/savior and rational/irrational:

Refugees, asylum seekers and undocumented migrants, especially women, infants and children, were identified as the most vulnerable ones. (p. 49)

As a ‘value system, a technique of government and a practice’, humanitarianism is always already ‘inherent within the commitment to seeing humanity’ (Pallister-Wilkins 2016, 509). Yet, due to humanitarianism’s roots in the colonial history of the British Empire (Skinner and Lester 2012), the ‘human’ constructed as reference point is always racialized. As a result, those who are not seen as entirely human due to the power of coloniality (Howell and Richter-Montpetit 2019) are dehumanized and constructed as inferior, dangerous or savage, and thus not part of what needs to be saved and protected. In the case of the RAR 2016, the humanitarian frame consequently ‘produces particular lives that are worthy of being saved or sacrificed’, whilst excluding others along racialized and gendered lines (Pallister-Wilkins 2016, 509). As such, it functions not as the opposite of technocratic managerialism, but as a tool to create order amongst the colonized ‘Others’. Accusations against male migrants for deliberately exploiting their ‘own’ women and children as ‘shields’ against enforcement practices (Frontex 2016, 11, see quote on p. 8 of this article) fit this framework of masculinist protectionism whereby racialized masculinities, embodied by the smuggler or other potentially violent migrants, are constructed as ruthless and barbarian. They allow Western authorities to present themselves as saviors of racialized women and children, reproducing the colonial narrative of ‘white men saving brown women from brown men’ (Spivak 1993). While those who are feminized are portrayed as legitimate victims requiring saving, others, who are hyper-masculinized, are constructed as chaos to Europe’s order (Skinner and Lester 2012; Reid-Henry 2014). This allows for gendered and racialized self-representations of Frontex and border guards as heroic, white, male protectors not only of the feminized European continent, which is exploited by racialized masses, but also of the vulnerable feminized ‘Other’.

**Figure 3.**
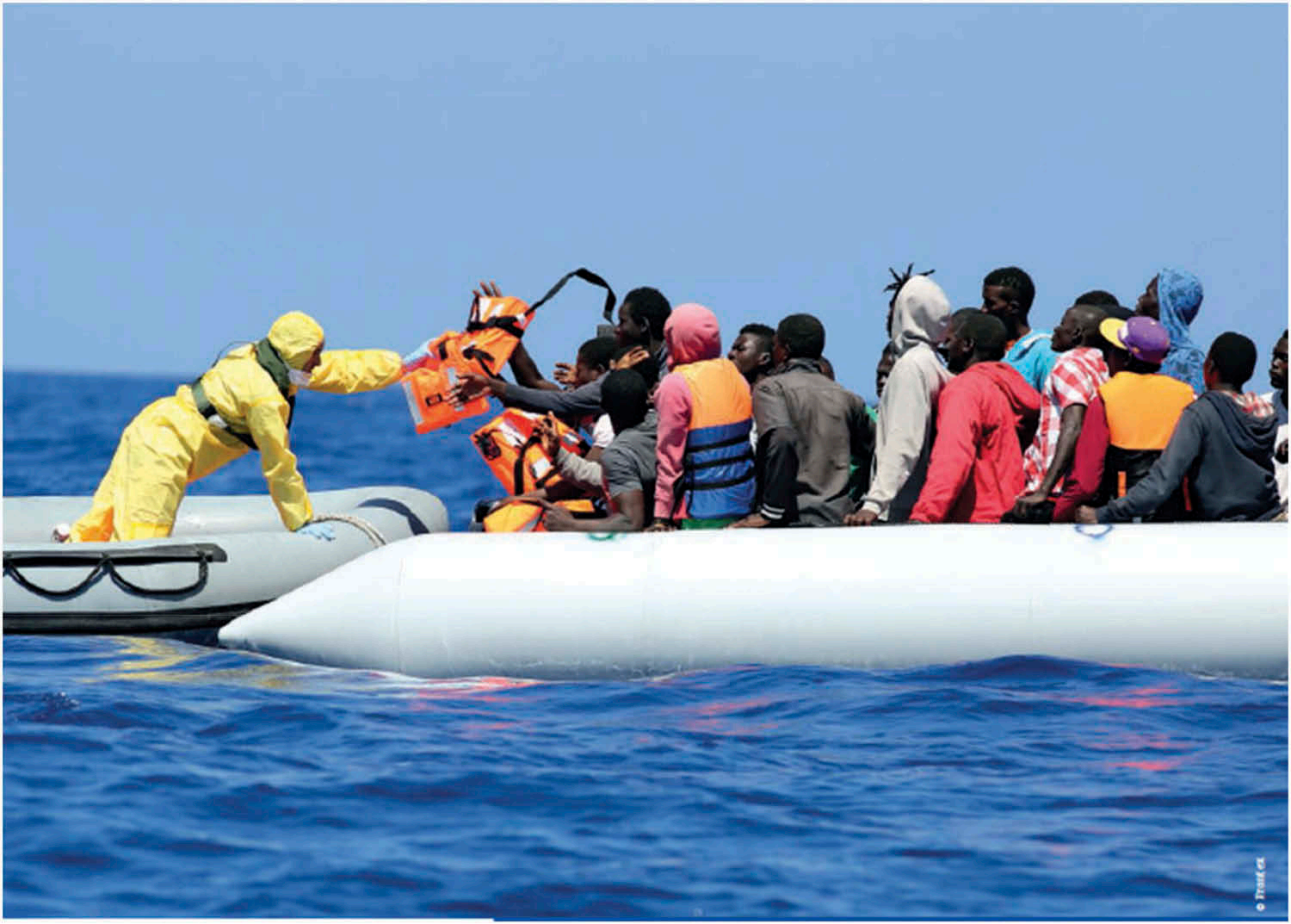
Rescue Operation, Frontex 2016, p. 7.

The analysis of the images in the RAR underscores these findings. The photographs give prevalence to the victim-savior narrative. Portrayals of border guards show male agents as rational and professional but at the same time benevolent and protective. They mostly appear in the role of tough and risk-taking protectors, e.g. pulling migrants aboard a ship (Frontex 2016, 7, 47). While images of ‘dramatic chaos’ at land borders frequently support the ‘womenandchildren’ trope of the written text, e.g. by depicting women taking care of small children while passively waiting at crowded and messy border checkpoints (p. 31), the larger, more prominent pictures, including the cover photo, depict sea rescues and draw an ambivalent image of the masculinized migrant as both a victim and a threat. These pictures show white, professional-looking men in clinical overalls or uniform-like attire assisting crowds of faceless, black men in colorful clothing. In one picture (see Figure 3; p. 47), an agent is handing out life vests to migrants on another boat. The black, male migrants are slightly struggling over the vests. This picture plays on the narratives of overstretch, chaos, and potential for violence associated with migration by producing a particularly gendered and racialized image of these migrants, while at the same time presenting Frontex as professional and humanitarian. Such portrayals speak to broader practices of international intervention ‘by those with the capacity and importantly the equipment to facilitate “safe” intervention’ (Pallister-Wilkins 2016, 508). Humanitarianism is thus ‘designed around the moral imperative to save lives and reduce suffering’ by using sophisticated tools and technologies (ibid.). While the RAR 2016 similarly highlights the importance of ‘technological fixes and technological fetishism’ (ibid.) to solve the (constructed) problems, its humanitarian discourse represents a form of managerialism of life that is deeply embedded in the condition of coloniality.

## 7. Conclusions

This article conducted an analysis of the gendered and racialized frames in Frontex’s RAR 2016, the centerpiece of the agency’s policy-relevant knowledge production. For this purpose, we brought a feminist postcolonial lens to security actors’ risk analysis, conceptualizing it as a sense-making practice that enables the (neo)colonial governing of migrants. From this perspective, we showed how gender and race underpin identified risk-definitions and proposed solutions, including the represented subjects of threat, victim, and protector in the context of coloniality. These frames build on and reify ‘Otherness’ by gendering and racializing migrants, their motivations, behavior, and choices and assign differential value, rights, and agency on the basis of perceived dichotomies between masculinity/femininity, (non-)/European, (non-)white, rational/irrational, civilized/chaotic, active/passive. Thereby, they create threat scenarios, which feminize European space as defendable territory (Pallister-Wilkins 2015), threatened to be penetrated by hypermasculinized and racialized intruders. In these scenarios, Frontex positions itself as the legitimate masculine protector of the European continent and the institutions and values at the heart of European identity, namely security and a functioning welfare system for the white European citizen. Only through proper management and a strengthened Frontex, it is argued, can these objectives be fulfilled; and only through the reproduction of migrants’ as the ‘Other’ can these approaches be made intelligible.

The suggested solutions build on these risk constructions, insisting on the need to know, categorize, manage, detain, and forcefully remove migrants. They require the gendered and racialized problematizations of the (masculinized) rational-but-immoral economic migrant, the ruthless and aggressive smuggler, and the (feminized) agency-less, naïve migrant to construct economic migration and people smuggling as root causes of migration. By producing migrants as both dangerous and vulnerable, these constructions constitute the rationale for deportations, detentions, anti-smuggling measures, Search and Rescue operations, increased surveillance, data sharing, and screening methods. Thereby, these frames not only disguise colonial power relations as well as the root causes of migration but also obscure the gendered and racialized risks and insecurities that border practices and policies entail for migrants.

The ways in which risks are framed, solutions suggested, and measures justified are embedded within gendered and racialized understandings of who/what constitutes a risk or a threat, which approaches are appropriate and efficient, and what ‘Europe’ and its values are in relation to the ‘Other’. Risk analysis is therefore not a neutral tool to assess threats and (in)securities but a sense-making security practice that makes migrants amendable to border management by Western authorities. Because Frontex’s risk analysis shapes policy-makers’ and public audiences’ understandings of migration and is continuously inscribed into Frontex’s operational cycle, this form of sense-making likely has wide-ranging consequences for border politics and practices. While these broader consequences are beyond the scope of this paper, gendered and racialized risk constructions in terms of unknownness and ‘Otherness’ might be at the bottom of problematic practices that inform contemporary bordering. These findings question whether increased insecurities for migrants can be addressed by anti-discrimination and gender mainstreaming efforts, when the nexus between gender inequality, (post)coloniality, and border security practices run so deeply within the perception of risks, actors’ roles, and self-identities.

Building on these results, further research is needed to clarify how gendered and racialized conceptualizations of (in)security and risk inform the process of data gathering and analysis in risk assessments; how the identified framings are formed in the process of risk analysis and how they translate into the conduct of security operations; and whether there are significant differences regarding the evolvement of gendered and racialized risk analysis since the foundation of Frontex in 2004. Our in-depth study of the RAR 2016 builds a foundation for this research by offering a theorization of risk analysis sensitive to gendered and racialized power relations. Bringing feminist and postcolonial scholarship to critical security studies on power and knowledge at/on the border thus contributes to a better understanding of how risk analysis ‘works’ by highlighting the ways in which it is complicit in reproducing sexist and colonial power relations that potentially lead to increased insecurities for migrants at the border.
